# MCEEGNet: a multi-cue EEG network for quantitative assessment of depression using emotional stimuli-induced EEG signals

**DOI:** 10.3389/frai.2026.1823559

**Published:** 2026-05-08

**Authors:** Xinmin Ding, Xingyu Chen, Minpeng Xu, Yuanmin Zhang, Hongli Chang

**Affiliations:** 1 Academy of Medical Engineering and Translational Medicine, Tianjin University, Tianjin, China; 2 School of Computer Science, Chengdu University of Information Technology, Chengdu, China; 3 China Unicom (Sichuan) Industrial Internet Co., Ltd., Chengdu, China; 4 Institute of Brain Science and Brain-inspired Research, Shandong First Medical University & Shandong Academy of Medical Sciences, Jinan, China

**Keywords:** depression assessment, EEG signals, emotional stimuli, Multi-Cue EEG network, quantitative evaluation

## Abstract

Depression significantly affects health, manifesting as alterations in typical emotional responses. Its diagnosis depends on subjective evaluations by clinicians, which are often time-intensive. Electroencephalogram (EEG) signals offer a viable solution for aiding diagnosis through computational means. However, current methods primarily focus on binary classification of depression, neglecting the quantification of depression risk. We propose the Multi-Cue EEG Network (MCEEGNet), which consists of parallel branches of EEGNet that extract features from various emotional stimuli to approximate scores on the Patient Health Questionnaire (PHQ-9). MCEEGNet aims to identify depression in patients using EEG signals and assess the severity of the condition. Our method achieved 91.13% accuracy in classification and reported Mean Squared Error (MSE) and Mean Absolute Error (MAE) of 20.45 and 3.28, respectively, in the Multi-Emotion Induced EEG Depression Database. The experimental outcomes suggest that MCEEGNet is highly effective in diagnosing subthreshold depression, offering a comprehensive system for evaluating clinical depression through EEG analysis influenced by multiple emotional cues, thereby meeting the need for quantitative depression evaluation.

## 1 Introduction

Depression represents a significant psychological disorder characterized by complex interactions with emotional states (Bullis et al., 2019). Those afflicted with depression commonly face difficulties in regulating their emotions, coupled with a reduced capacity to respond to routine emotional experiences (Denecke et al., 2020). The compromised quality of life experienced by individuals with depression renders it a critical risk factor for suicide (Turecki et al., 2019). Unfortunately, the dearth of effective treatment approaches and the limited availability of mental health resources contribute to the frequent underdiagnosis and untreated nature of depression.

The pursuit of reliable biomarkers for diagnosing depression is of significant importance for enhancing the treatment of neuropsychiatric disorders (de Aguiar Neto and Rosa, 2019). In recent years, non-invasive tools, such as Electroencephalography (EEG), have gained widespread application in research (Liu et al., 2022; Chang et al., 2022, 2023). A noteworthy research endeavor involves the development of efficient neural network-based methods for analyzing EEG signals, enabling automated depression assessment. EEG, being a non-invasive, efficient, and potent tool, has been utilized to record brain electrical activities associated with various conditions, including Parkinson's disease (Chang et al., 2023), Alzheimer's disease (Gaubert et al., 2019), and sleep disorders (Dimitriadis et al., 2021).

Depression exerts an impact on neural signaling within the brain, leading to altered brain activity patterns (Needham et al., 2020). This alteration manifests as a slowing down of specific brain functions due to the transmission of signals from the body to the brain, thereby influencing the generation and communication of neurons. Consequently, voltage changes produced by ion currents within brain neurons hold promise for diagnosing mental disorders, such as depression. However, the complexity, lack of structure, and substantial variations linked to individuals, age, and mental states pose challenges and complexities in the development of robust methods for analyzing brain signals (Stern et al., 2019). Additionally, EEG signals are often subject to various types of noise, such as eye blinks and body movements (Kilicarslan et al., 2016), necessitating the utilization of deep learning techniques capable of effectively extracting brain activity patterns from EEG signals.

In the field of depression research, the event-related potential (ERP) elicited by various experimental paradigms involving multiple emotional stimuli holds significant importance (Bylsma, 2021). Individuals dealing with depression often face challenges related to emotion regulation. By utilizing different types of emotional stimuli in a controlled laboratory setting, researchers can gain deeper insights into changes in EEG activity across various emotional states among patients. On the one hand, depression is a complex psychological disorder. The use of multiple emotional stimuli provides a more comprehensive understanding of how a patient's EEG activity evolves across different emotional states, enabling a more profound exploration of the underlying mechanisms of depression (Bigdeli et al., 2017; Durisko et al., 2015).

On the other hand, different patients with depression may exhibit varying responses to different emotional stimuli (Bos et al., 2019). Some individuals may show significantly heightened EEG activity when exposed to negative emotional stimuli, while others may display distinct EEG responses to positive emotional stimuli. As a result, the inclusion of multiple stimuli is invaluable in capturing the range of interindividual differences. Additionally, the treatment of depression may involve interventions such as emotion regulation training. Understanding how EEG patterns change in response to diverse emotional states can provide valuable insights for tailoring personalized treatment strategies (Hajal and Paley, 2020). Therefore, in scientific inquiry, the incorporation of diverse stimulus conditions is essential to ensure the reliability and generalizability of research findings (Woo et al., 2017). Taking multiple emotional stimuli into account enhances the accuracy of identifying EEG activity patterns associated with depression.

Previous studies have exclusively examined EEG cues elicited by a single type of emotional stimulus. There exists a divergence of perspectives: some assert that depression-associated emotions exhibit a negative bias (Chang et al., 2023; Li et al., 2018), while others contend that positive emotional stimuli are more readily discerned (Liu et al., 2022; Chang et al., 2022). Additionally, some methods aggregate all cues indiscriminately, feeding them into the model for collective learning. While this approach augments the sample size, it often leads to a deterioration in the recognition performance of the learned model, resulting in a “1+1 < 2” phenomenon (Chang et al., 2023; Li et al., 2018; Liu et al., 2022; Chang et al., 2022). Hence, the incorporation of various emotional cues in the acquisition of EEG signals in depression research assumes critical importance. This practice not only contributes to the elucidation of the neural mechanisms underlying depression and individual distinctions but also furnishes valuable insights for advancing our comprehension and treatment of depression.

The severity of depression is typically assessed using the PHQ-9 scale test, which yields scores ranging from 0 to 27. Due to the significant variations and spans in individual scores, regression problems may encounter challenges. In such cases, the model may exhibit heightened sensitivity to labels with larger values and diminished sensitivity to those with smaller values (Ribeiro et al., 2018). Normalization serves to homogenize all labels into a relatively uniform range. Additionally, when label values span a wide range, the model may require prolonged adjustment of weights during training to accommodate these extensive label ranges, potentially impeding convergence. Normalization expedites convergence by obviating the need for continuous weight adjustments across disparate label scales. Furthermore, specific activation functions (e.g., sigmoid, tanh) exhibit heightened sensitivity within certain ranges. If label values extend beyond the sensitive range of the activation function, the model's predictive performance may be compromised. Normalization ensures that label values align appropriately with the activation function employed by the model (Peng et al., 2022). Lastly, in terms of gradient descent stability, a wide range of label values can lead to erratic gradient updates, thereby disrupting the model training process. Normalization mitigates this instability, fostering more stable model training (Pan et al., 2022). Consequently, normalizing data labels in regression prediction not only facilitates more effective learning of data relationships but also enhances training outcomes and prediction performance.

To tackle these challenges, we propose a novel end-to-end architecture, Multi-Cue EEG Network (MCEEGNet), consisting of three parallel EEGNets (Liu et al., 2022; Lawhern et al., 2018), each extracting EEG features of different cues, designed to extract meaningful features from EEG data effectively, obviating the need for high-complexity EEG preprocessing. By leveraging the advantages of multiple cues, this approach enhances the accuracy, comprehensiveness, and precision of depression diagnosis. Notably, MCEEGNet takes raw EEG signals as input, which are filtered through specific frequency bands to eliminate high and low-frequency artifacts. In this study, EEG signals were collected from 53 individuals using 128 scalp electrodes (Cai et al., 2020a; Hu et al., 2017; Li et al., 2018). All participants underwent Patient Health Questionnaire (PHQ-9) testing (Kroenke et al., 2001), and their scores were determined. MCEEGNet achieved a classification accuracy of 91.13%. For regression tasks, it yielded a mean squared error (MSE) of 20.45 and a mean absolute error (MAE) of 3.28. Experimental results demonstrated that MCEEGNet surpassed baseline methods, exhibited robustness in feature extraction, and outperformed them in classification outcomes and regression predictions.

MCEEGNet adeptly extracts features from EEG data while continually estimating PHQ-9 scores in individuals with depression. This methodology proves applicable not only to classification tasks but also to regression tasks, owing to the diverse numerical ranges of PHQ-9 assessment metrics. The normalization of data labels amplifies model performance and enhances training efficacy. Comprehensive experimental findings attest to MCEEGNet's excellence in depression diagnosis, manifesting in high classification accuracy and regression prediction accuracy that surpass existing methods. This research introduces a novel perspective to biomarker studies for depression and holds the potential to advance more precise diagnostics and personalized treatment strategies in future clinical practice.

The main contributions of this study are as follows:

(1) We comprehensively consider EEG responses elicited by different emotional stimuli and propose MCEEGNet, which extracts stimulus-specific features through separate branches, thereby alleviating the “1 + 1 < 2” problem in naive multi-cue fusion for depression assessment.

(2) Beyond binary depression classification, MCEEGNet enables continuous score assessment by directly predicting the PHQ-9 score on its full numeric scale through a regression framework. This design allows the model to quantify depression severity rather than merely outputting a categorical label, which is particularly useful for identifying subthreshold depression.

(3) For the continuous PHQ-9 score prediction task, we further demonstrate that label normalization improves optimization stability and predictive accuracy, leading to more reliable regression performance across subjects with different severity levels.

The remainder of this paper is organized as follows: Section II outlines our methodology, including research design, experimental setup, etc.; Section III provides a thorough analysis of the experimental results, and discusses the implications of our findings; and finally, Section IV concludes the paper with a summary of key takeaways and suggestions for future research directions.

## 2 Methodology

We utilized the MODMA dataset,^1^ comprising 128-channel Event-Related Potentials (ERPs) recorded from 24 patients diagnosed with major depressive disorder and 29 healthy control individuals. Participant ages ranged from 16 to 52 years, and a summary of participant information is available in Table 1. The data were recorded at a sampling rate of 250 Hz and encompassed a dot-probe task featuring happy-neutral (“happy cue”), fearful-neutral (“fear cue”), and sad-neutral (“sad cue”) face pairs. Participants were seated at a distance of 60 cm from the monitor and were instructed to press a button upon the appearance of a dot on either side of a fixed crosshair following the presentation of emotion-neutral face cues lasting 500 milliseconds. The experiment was divided into three sections, each comprising 160 trials. Each trial commenced with a stationary white crosshair and had a duration of approximately 25 min (Cai et al., 2020a; Hu et al., 2017; Li et al., 2018).

**Table 1:** MODMA dataset subject information table.

Subject ID	Type	Gender	PHQ-9	Subject ID	Type	Gender	PHQ-9
1	MDD	F	23	28	HC	F	3
2	MDD	F	12	29	HC	F	0
3	MDD	M	19	30	HC	M	3
4	MDD	M	16	31	HC	M	3
5	MDD	M	17	32	HC	M	3
6	MDD	M	19	33	HC	M	5
7	MDD	M	24	34	HC	M	4
8	MDD	F	22	35	HC	M	3
9	MDD	F	11	36	HC	M	0
10	MDD	M	14	37	HC	M	2
11	MDD	F	18	38	HC	M	2
12	MDD	M	19	39	HC	M	4
13	MDD	F	21	40	HC	M	5
14	MDD	F	20	41	HC	M	0
15	MDD	M	18	42	HC	M	5
16	MDD	F	16	43	HC	F	3
17	MDD	F	23	44	HC	F	0
18	MDD	M	20	45	HC	F	0
19	MDD	M	15	46	HC	F	0
20	MDD	M	17	47	HC	M	4
21	MDD	F	24	48	HC	F	3
22	MDD	F	19	49	HC	M	3
23	MDD	M	16	50	HC	M	3
24	MDD	M	17	51	HC	M	4
25	HC	M	1	52	HC	F	5
26	HC	M	4	53	HC	F	0
27	HC	M	5				

**Figure 1:**
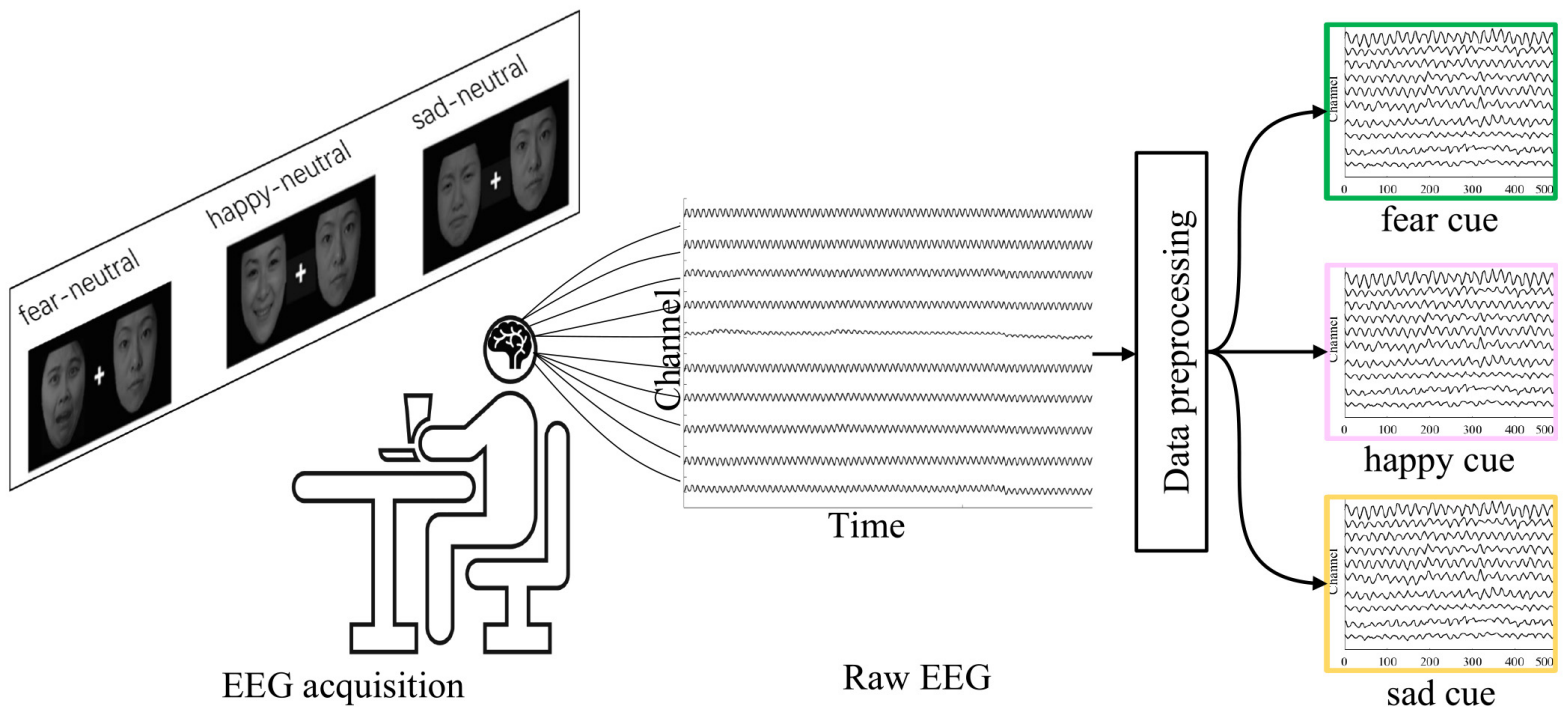
EEG pre-processing pipeline. The raw EEG signals were successively processed by average re-referencing, band-pass filtering, event-related epoch extraction, baseline correction, ICA-based artifact decomposition, and automatic artifact component identification using the ADJUST algorithm, followed by removal of noise-related components.

The pre-processing of EEG data was performed using MATLAB and the EEGLAB toolbox^2^ (Brunner et al., 2013), and it involved the following steps (Liu et al., 2022; Chang et al., 2023): Transformation of EEG data to the average reference; Application of a bandpass filter to the data (0.3–100 Hz) to eliminate 50 Hz power line interference; Segmentation of EEG data into event-locked epochs corresponding to “hcue,” “fcue,” and “scue”; Removal of the mean baseline (–100 to 0) from the segmented EEG; Application of Independent Component Analysis (ICA) decomposition, with specified components removed; Utilization of the Adjust algorithm for subtracting component activity (Mognon et al., 2011). The results of EEG data preprocessing are illustrated in Figure 1.

The resulting preprocessed data was structured as [trials, channels, samples, kernels], with the following dimensions: *trials* = 480, *channels* = 128, *samples* = 125, and *kernels* = 1. For the classification model, normal samples were labeled as 0, while severe depression samples were labeled as 1. In the case of the regression prediction model, the labels for all samples of each participant were derived from their PHQ-9 psychological health questionnaire scores. For the regression task, the labels for all samples from the same subject were assigned according to that subject's PHQ-9 score. Following the conventional PHQ-9 severity categorization, scores of 0–4, 5–9, 10–14, 15–19, and 20–27 correspond to minimal, mild, moderate, moderately severe, and severe depression, respectively. In this study, the severe depression refers to subjects with PHQ-9 scores≥20. No additional exclusion threshold was imposed beyond the availability of valid EEG recordings and PHQ-9 annotations in the dataset.

The normalization of data labels is a crucial step that aids the model in learning data relationships more effectively, leading to improved training efficiency and predictive performance. We employed subject-wise min-max normalization on the PHQ-9 labels, where the score range of each subject was linearly mapped to [0, 1]. This normalization process ensures that the data labels fall within a consistent and interpretable range, facilitating effective model training and interpretation.

### 2.1 MCEEGNet

**Figure 2:**
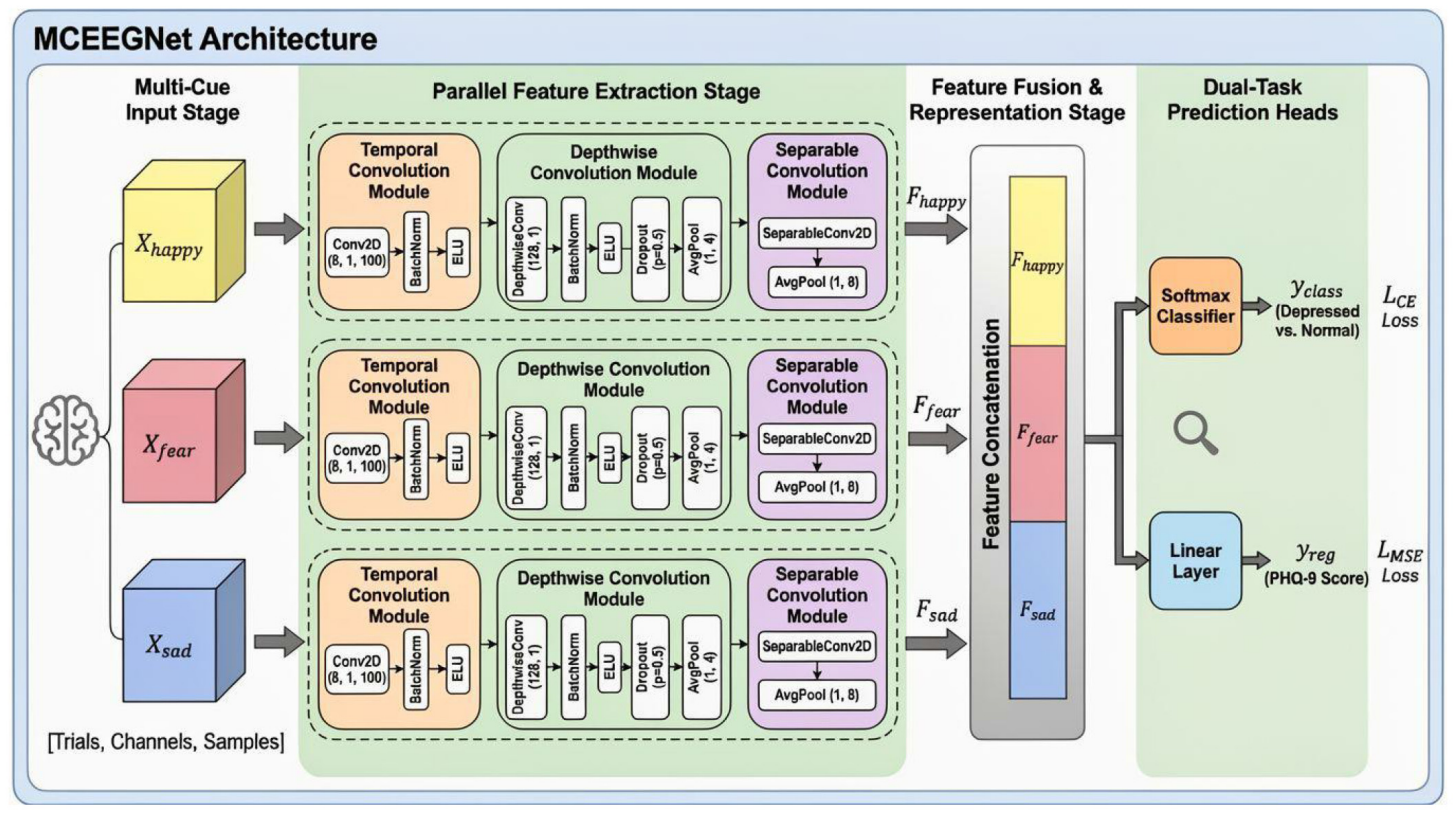
Framework of the proposed MCEEGNet.

To effectively model EEG signals induced by multiple emotional stimuli and quantitatively assess the severity of depression, this study proposes a Multi-Cue EEG Network (MCEEGNet). The model consists of three parallel EEGNet branches (Lawhern et al., 2018), each responsible for processing EEG signals under different emotional conditions: happy cue, fear cue, and sad cue, as illustrated in Figure 2. This design enables multi-perspective feature extraction and fusion.

Temporal Convolution Module: Eight 1 × 100 2D convolutional filters are used to extract frequency-related features from the input EEG signal (Ioffe and Szegedy, 2015),


$$\begin{array}{l} X_{1}={\text{Conv2D}}_{\left(1,100\right)}\left(X\right),\;X_{1}\in {\mathbb{R}}^{8\times C\times T^{\prime }} \end{array}$$ (1)

Batch normalization and the Exponential Linear Unit (ELU) activation function are then applied (Clevert et al., 2015),


$$\begin{array}{l} X_{1}=\text{BatchNorm}\left(X_{1}\right),\;X_{1}=\text{ELU}\left(X_{1}\right) \end{array}$$ (2)

Depthwise Convolution Module: A depthwise convolution with kernel size 128 × 1 is used to extract spatial features and reduce the number of trainable parameters,


$$\begin{array}{l} X_{2}={\text{DepthwiseConv}}_{\left(C,1\right)}\left(X_{1}\right) \end{array}$$ (3)

This is followed by batch normalization, ELU activation, dropout (with *p* = 0.5), and average pooling with a kernel size of 1 × 4,


$$\begin{array}{l} X_{2}={\text{AvgPool}}_{\left(1,4\right)}\left(\text{Dropout}\left(\text{ELU}\left(\text{BatchNorm}\left(X_{2}\right)\right)\right)\right) \end{array}$$ (4)

Separable Convolution Module: A 1 × 16 depthwise separable convolution is then used to integrate temporal features efficiently,


$$\begin{array}{l} X_{3}=\text{SeparableConv2D}\left(X_{2}\right) \end{array}$$ (5)

An average pooling layer with a size of 1 × 8 is then applied for dimensionality reduction.

Feature Fusion and Output: The feature maps from the three branches are flattened and concatenated to form a combined multi-cue representation, which is then fed into classification or regression heads.

Each branch receives EEG data corresponding to one emotional condition, denoted as **X**_happy_, **X**_fear_, **X**_sad_, and outputs high-level spatiotemporal features through independent processing:


$$\begin{array}{l} {\text{F}}_{\text{happy}}=\text{EEGNet}\left({\text{X}}_{\text{happy}}\right) \end{array}$$ (6)

$$\begin{array}{l} {\text{F}}_{\text{fear}}=\text{EEGNet}\left({\text{X}}_{\text{fear}}\right) \end{array}$$ (7)

$$\begin{array}{l} {\text{F}}_{\text{sad}}=\text{EEGNet}\left({\text{X}}_{\text{sad}}\right) \end{array}$$ (8)

These feature vectors are then concatenated to form a unified multi-cue representation:


$$\begin{array}{l} {\text{F}}_{\text{concat}}=\text{concat}\left({\text{F}}_{\text{happy}},{\text{F}}_{\text{fear}},{\text{F}}_{\text{sad}}\right) \end{array}$$ (9)

MCEEGNet is designed to simultaneously perform depression classification and PHQ-9 score regression. For classification tasks, a softmax classifier is used to output the probability of each class (depressed or normal):


$$\begin{array}{l} {ŷ}_{\text{class}}=\text{Softmax}\left({\text{W}}_{\text{cls}}\cdot {\text{F}}_{\text{concat}}+{\text{b}}_{\text{cls}}\right) \end{array}$$ (10)

For regression tasks, a fully connected linear layer outputs a normalized PHQ-9 prediction:


$$\begin{array}{l} {ŷ}_{\text{reg}}={\text{W}}_{\text{reg}}\cdot {\text{F}}_{\text{concat}}+{\text{b}}_{\text{reg}} \end{array}$$ (11)

To improve regression performance and training stability, label normalization using min-max scaling is applied:


$$\begin{array}{l} y^{*}=\frac{y-y_{\min}}{y_{\max}-y_{\min}} \end{array}$$ (12)

During inference, the predicted values are rescaled to the original PHQ-9 range via inverse normalization:


$$\begin{array}{l} y=y^{*}\cdot \left(y_{\max}-y_{\min}\right)+y_{\min} \end{array}$$ (13)

During training, the cross-entropy loss function is used for classification tasks:


$$\begin{array}{l} L_{\text{CE}}=-\overset{N}{\underset{i=1}{\sum }}\overset{C}{\underset{j=1}{\sum }}y_{ij}\log{ŷ}_{ij} \end{array}$$ (14)

and the mean squared error (MSE) is adopted for regression:


$$\begin{array}{l} L_{\text{MSE}}=\frac{1}{N}\overset{N}{\underset{i=1}{\sum }}{\left(y_{i}-{ŷ}_{i}\right)}^{2} \end{array}$$ (15)

The model is optimized using the Adam optimizer with 20 training epochs. A validation set comprising 10% of the data is used, and early stopping is employed to retain the model weights corresponding to the lowest validation loss. This multi-cue architecture significantly enhances the model's ability to capture depression-related EEG patterns under various emotional conditions and mitigates the 1+1 < 2 issue commonly encountered in naive feature fusion.

### 2.2 Evaluation metrics

Following the modeling phase, it becomes imperative to employ metrics for evaluating the model's generalization capacity and iteratively fine-tuning parameters to optimize its performance. The Confusion Matrix, a vital tool in machine learning and statistics, serves as an instrument for assessing the performance of classification models, particularly in gauging accuracy and misclassifications. It offers comprehensive insights into the classification model's performance by examining the alignment of true and predicted class labels.

When the model accurately identifies normal individuals as normal, it is classified as a True Positive (TP); conversely, when it erroneously categorizes them as depressed, it is labeled as a False Negative (FN). When the model correctly recognizes depressed patients as depressed, it is designated as a True Negative (TN); in contrast, if it incorrectly identifies them as normal, it is denoted as a False Positive (FP). Subsequently, the following five classification evaluation metrics are employed:

Accuracy is defined as the proportion of correctly classified samples among the total samples. This evaluation metric treats each class equally and is often used as a measure of overall model performance.


$$\begin{array}{l} \text{Accuracy}=\frac{TP+TN}{TP+TN+FP+FN} \end{array}$$ (16)

Precision is calculated as the proportion of correctly predicted positive samples out of all samples that were predicted as positive. This metric focuses on the accuracy of positive predictions and is particularly useful when the cost of false positives is high.


$$\begin{array}{l} \text{Precision}=\frac{TP}{TP+FP} \end{array}$$ (17)

Recall is defined as the proportion of correctly predicted positive samples out of all actual positive samples. This metric assesses the model's ability to identify all relevant instances of the positive class and is valuable when the cost of false negatives is high.


$$\begin{array}{l} \text{Recall}=\frac{TP}{TP+FN} \end{array}$$ (18)

F1-Score is a metric that strikes a balance between precision and recall, taking into account their weighted harmonic mean. It is particularly valuable for addressing the trade-off between these two metrics and for considering situations with class imbalances. The F1-Score provides a single value that reflects the model's performance in terms of both precision and recall, making it a useful overall evaluation metric for classification tasks.


$$\begin{array}{l} \text{F1-Score}=\frac{2\times Precision\times Recall}{Precision+Recall} \end{array}$$ (19)

Kappa Coefficient is an indicator utilized to measure consistency and assess classification performance. It quantifies the level of agreement between model predictions and actual class labels. This metric provides valuable insights into the model's classification performance, accounting for the possibility of agreement occurring by chance. It is particularly useful in situations where class imbalances or random agreement might affect other evaluation metrics.


$$\begin{array}{l} \text{Kappa}=\frac{P_{a}-P_{e}}{1-P_{e}}, \end{array}$$ (20)

$$\begin{array}{l} P_{a}=\frac{TP+TN}{TP+TN+FP+FN}, \end{array}$$ (21)

$$\begin{array}{l} P_{e}=\frac{\left(TP+FP\right)\left(TP+FN\right)+\left(FN+TN\right)\left(FP+TN\right)}{{\left(TP+TN+FP+FN\right)}^{2}} \end{array}$$ (22)

These metrics encompass a range of values within the [0, 1] interval, where a higher value signifies superior predictive performance for the model. Through the utilization of the confusion matrix in conjunction with these metrics, we can conduct a comprehensive assessment of the model's performance across various scenarios. For instance, a scenario characterized by high accuracy but low recall might suggest that the model excels in classifying the negative class while missing some samples within the positive class. Conversely, in cases of high recall but low precision, it may indicate that the model is sensitive to the positive class but may also misclassify certain samples from the negative class. These metrics play a pivotal role in furnishing a deeper understanding of the model's classification capabilities and identifying potential issues.

In the context of regression models, where there are *n* samples, each sample is represented as (*x*_*i*_, *y*_*i*_), with corresponding predictions denoted as ŷ_*i*_ for *i*∈{1, 2, …, *n*·ȳ}, where ȳ signifies the average of ${\left\{y_{i}\right\}}_{i=1}^{n}$.

Mean Squared Error (MSE) corresponds to the expectation of the squared (second-order) error.


$$\begin{array}{l} \mathrm{MSE}\left(\text{y},\text{ŷ}\right)=\frac{1}{n}\overset{n}{\underset{i=1}{\sum }}||y_{i}-{ŷ}_{i}|{|}_{2}^{2} \end{array}$$ (23)

Root Mean Squared Error (RMSE) is the square root of the average of the squared differences between the predicted values and the actual observed values. RMSE gives more weight to larger errors, making it sensitive to outliers.


$$\begin{array}{l} \mathrm{RMSE}\left(\text{y},\text{ŷ}\right)=\sqrt{\frac{1}{n}\overset{n}{\underset{i-1}{\sum }}||y_{i}-{ŷ}_{i}|{|}_{2}^{2}} \end{array}$$ (24)

Mean Absolute Error (MAE) is the average of the absolute differences between the predicted values and the actual observed values. It represents the average of the absolute errors, disregarding their direction, and is therefore relatively less sensitive to outliers.


$$\begin{array}{l} \mathrm{MAE}\left(\text{y},\text{ŷ}\right)=\frac{1}{n}\overset{n}{\underset{i=1}{\sum }}|y_{i}-{ŷ}_{i}| \end{array}$$ (25)

Median Absolute Error (MEDAE) is the median of the absolute differences between the predicted values and the actual observed values. The median is less affected by outliers, so when there are many outliers present, MEDAE may be more robust and stable compared to other error metrics.


$$\begin{array}{l} \mathrm{MEDAE}\left(\text{y},\text{ŷ}\right)=\mathrm{median}\left(\left|y_{1}-{ŷ}_{1}|,\ldots ,|y_{n}-{ŷ}_{n}\right|\right) \end{array}$$ (26)

These regression metrics are reported together because they capture complementary aspects of prediction error. MSE and RMSE assign greater penalties to large deviations and are therefore more sensitive to outliers, making them useful for evaluating whether a model occasionally produces large prediction errors. In contrast, MAE reflects the average absolute deviation in a more directly interpretable manner, while MEDAE is more robust to extreme values and better reflects the typical prediction error across subjects. Reporting all four metrics provides a more comprehensive assessment of regression performance.

In this study, continuous score assessment refers to predicting each subject's PHQ-9 value as a continuous numerical target using regression, rather than assigning only a binary class label (depressed vs. normal). This setting allows the model to estimate symptom severity on the full PHQ-9 scale and supports finer-grained evaluation of depression risk.

Indeed, these four metrics, namely MSE, RMSE, MAE, and MEDAE, serve as critical tools for evaluating the performance of regression models. Smaller values of these metrics indicate more accurate predictions. For instance, if a model exhibits a relatively small RMSE, it signifies that it offers accurate predictions across the dataset. A comparative analysis of these metrics can swiftly provide insights into the model's performance on the dataset.

## 3 Results and discussion

### 3.1 Results on the classification recognition task

**Figure 3:**
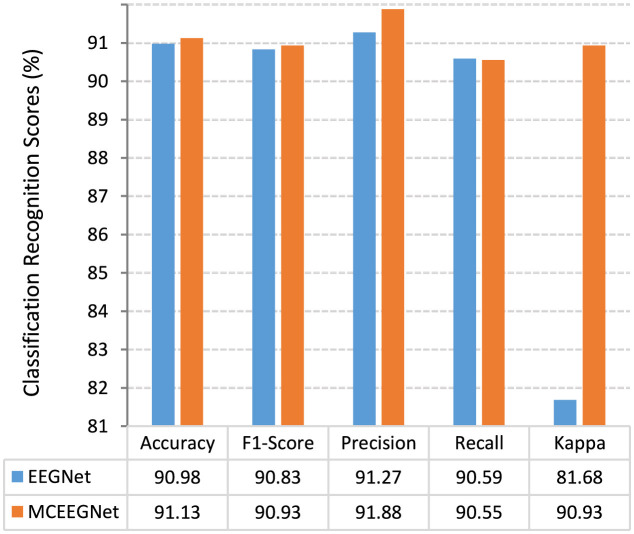
Comparison of classification and recognition scores between MCEEGNet and EEGNet.

MCEEGNet incorporates the EEG responses evoked by three distinct emotional stimuli to decode depression, resulting in the generation of five recognition scores, as illustrated in Figure 3. Upon closer examination of the figure, it becomes evident that, except recall, all other scores demonstrate improvements when compared to the best result achieved by EEGNet under the “hcue” condition. Notably, the Kappa coefficient exhibits a substantial increase of nearly 10%. This noteworthy enhancement underscores the significant improvement in recognition performance achieved through the fusion of features extracted from the three emotional stimuli.

**Figure 4:**
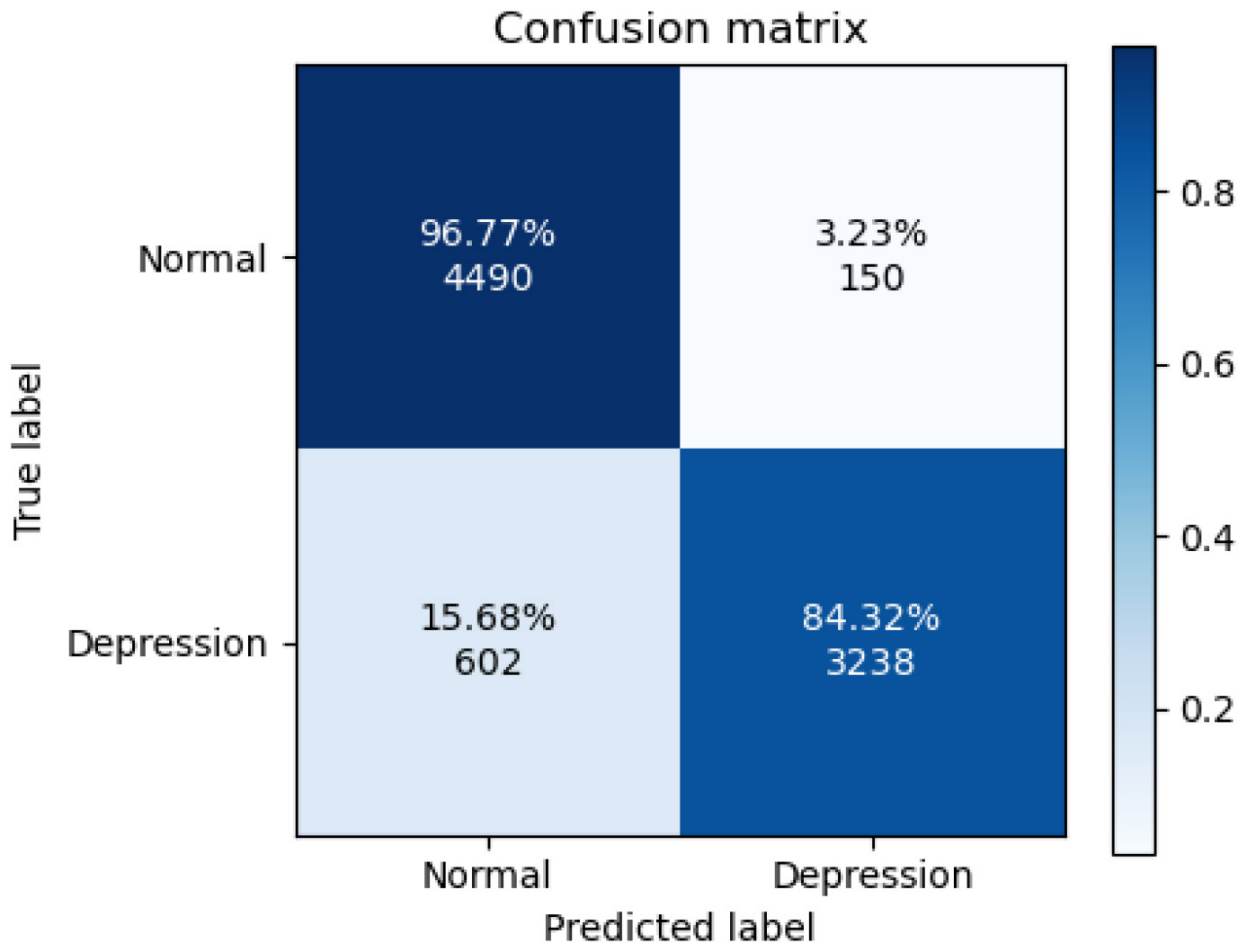
Confusion matrix of the proposed method on the classification recognition task.

Furthermore, the utilization of the confusion matrix provides a more detailed and comprehensive assessment of the classification model's performance. It serves as a valuable tool for understanding both the strengths and limitations of the model, guiding adjustments, and facilitating improvements in real-world applications, as exemplified in Figure 4. Notably, the recognition performance is most prominent for healthy subjects. This observation may be attributed to the dataset containing a larger number of samples from healthy subjects, enabling the model to gain a more profound understanding of the characteristics associated with the normal class.

#### 3.1.1 Comparison with existing methods

Owing to disparities in methods, datasets, and strategies for data utilization, conducting a comprehensive assessment of the strengths and weaknesses of various approaches based solely on classification accuracy presents challenges. Nonetheless, the comparison of metrics, such as accuracy, can offer partial or indirect insights into the merits and drawbacks of different methods. In light of this consideration, Table 2 furnishes a comprehensive comparison of existing state-of-the-art methods and our proposed approach. This comparison encompasses key attributes, including the number of subjects, types, and quantities of channels, research methodologies, feature quantities, and classification accuracy.

**Table 2:** Comprehensive comparison of existing state-of-the-art methods with the proposed method.

Method	Subject (MDD,HC)	Channel	Feature	Protocol	Accuracy
Independent component analysis (Zhang et al., 2018)	(13, 13)	64	-	-	-
SVM (Chang et al., 2023)	(24, 29)	128	640 EEG	LOSOCV	41.77
KNN (Chang et al., 2023)	(24, 29)	128	640 EEG	LOSOCV	54.47
BLDA (Chang et al., 2023)	(24, 29)	128	640 EEG	LOSOCV	61.37
Random Forest (Chang et al., 2023)	(24, 29)	128	640 EEG	LOSOCV	65.83
EEGNet (Chang et al., 2023)	(24, 29)	128	640 EEG	LOSOCV	70.46
Spatio-temporal neural network (Chang et al., 2022)	(24, 29)	128	640 EEG	Five-fold CV	71.14
EEGNet (All trials) (Liu et al., 2022)	(24, 29)	128	Raw EEG	LOSOCV	78.46
KNN (Cai et al., 2018)	(92, 121)	3	270 EEG	Ten-fold CV	79.27
Convolutional Neural Network (Li et al., 2019)	(24, 27)	128	Spectral, Spatial	24-fold CV	85.62
EEGNet (Fcue trials) (Liu et al., 2022)	(24, 29)	128	Raw EEG	LOSOCV	86.00
EEGNet (Scue trials) (Liu et al., 2022)	(24, 29)	128	Raw EEG	LOSOCV	86.20
Multimodal fusion (Zhang et al., 2019)	(81, 89)	4	6 EEG, 15 voice	Nested CV	86.64
Feature-level fusion (Cai et al., 2020b)	(86, 92)	3	60 linear, 36 non-linear	Ten-fold CV	86.98
Improved Empirical Mode Decomposition (Shen et al., 2019)	(105, 70)	3	4 feature vectors	Ten-fold CV	88.07
Correlated Feature Selection (Li et al., 2018)	(17, 17)	128	10 EEG	LOSOCV	88.94
SVM (Liu et al., 2020)	(20, 19)	64	3 potential biomarker	Ten-fold CV	89.70
EEGNet (Hcue trials) (Liu et al., 2022)	(24, 29)	128	Raw EEG	LOSOCV	90.98
MCEEGNet (ours)	(24, 29)	128	Raw EEG	LOSOCV	91.13

Our approach presents significant advantages when compared to other methods. Firstly, considering the dataset perspective, methods like Feature-level Fusion (Cai et al., 2020b), KNN (Cai et al., 2018), and our approach all contend with class imbalance, denoting a substantial difference in the number of subjects between the MDD and HC categories. Notably, our approach attains the highest accuracy. Class imbalance can often result in models performing well in the larger class while struggling with the smaller one.

Secondly, about the testing protocol, since depression classification is inherently subject-specific, Leave-One-Subject-Out Cross-Validation (LOSOCV) proves to be a more suitable choice than ten-fold cross-validation. LOSOCV ensures that data from the same subject are kept together, preventing the scenario where some data from the same subject end up in the training set while others are in the test set. Such a situation could potentially mislead the classifier into recognizing the subject's identity rather than depression, thereby inflating recognition performance. Therefore, after considering data balance and testing protocol analysis, the proposed method emerges as achieving the best recognition performance.

Moreover, when compared to Multimodal Fusion (Zhang et al., 2019), our approach exhibits approximately a 5% higher accuracy, eliminates the need for manual feature extraction, and effectively addresses sample imbalance.

In comparison to EEGNet (Liu et al., 2022), a method utilizing the same dataset, our approach exhibits superior performance when individual types of stimuli are considered independently. Conversely, EEGNet's recognition performance diminishes when multiple types of stimulus trials are simultaneously input to the model. This phenomenon can be attributed to increased interference information as the number of samples grows, leading to a “1+1 < 2” scenario. MCEEGNet, by employing three separate branches to extract features from distinct types of stimuli, excels in capturing features associated with depression more effectively, thereby yielding enhanced depression recognition.

### 3.2 Results on the regression prediction task

**Figure 5:**
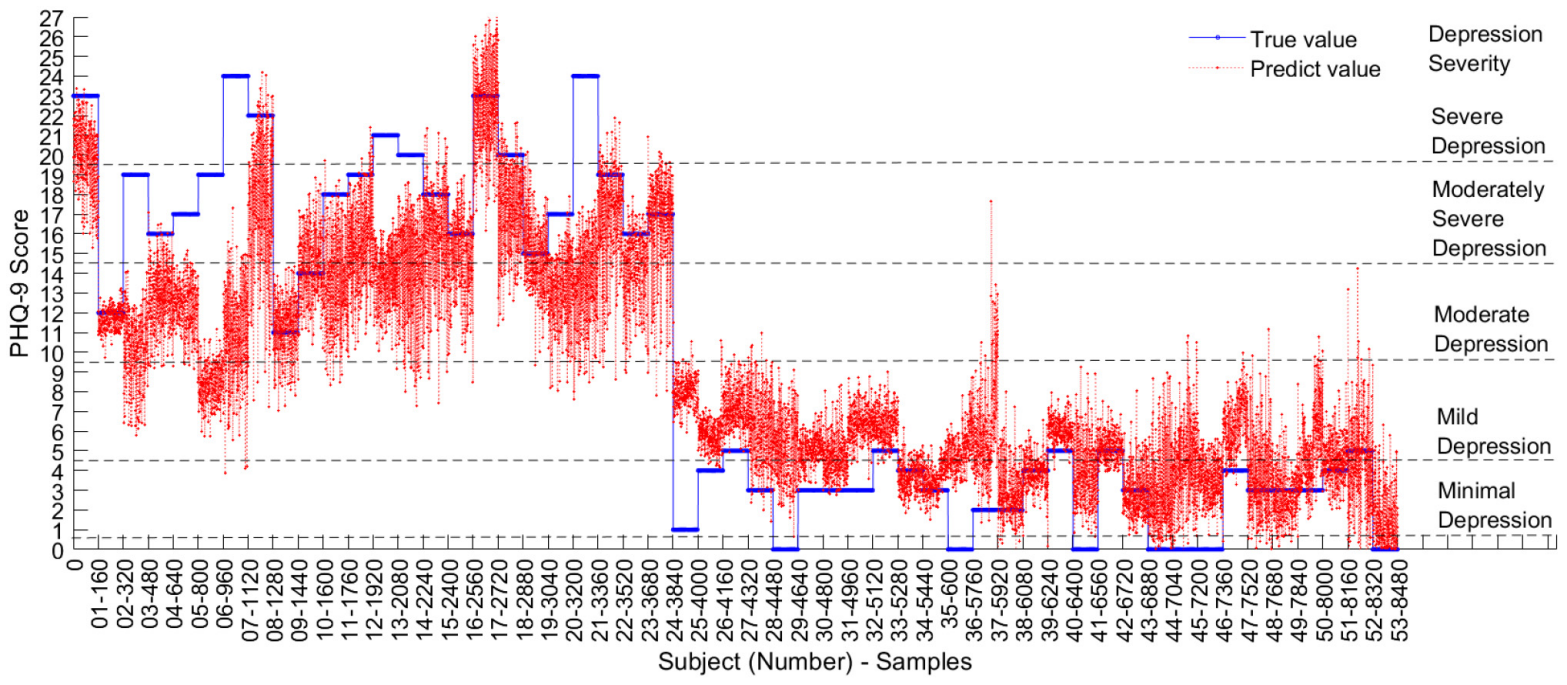
MCEEGNet prediction results in regression prediction.

Figure 5 visually presents the prediction outcomes of MCEEGNet for each sample in depression regression. The horizontal axis organizes the samples by subject IDs, while the vertical axis represents the PHQ-9 scores. The blue horizontal lines correspond to the actual scores of each subject, whereas the red lines depict the scores predicted by the model. Notably, on the extreme right, the severity of depression is categorized into five distinct levels based on the total scores.

For most subjects, the predicted values are in close agreement with the true depression severity levels, particularly for subjects with mild-to-moderate symptoms. However, subjects 3, 6, 7, 21, and 25 show relatively larger prediction errors, with the predicted values differing by approximately one severity level from the ground truth. Several factors may account for this discrepancy. First, these subjects may represent relatively difficult or atypical cases, whose EEG patterns deviate from the dominant patterns learned by the model. Second, the PHQ-9 labels of some subjects may lie near category boundaries, making small numerical deviations more likely to translate into severity-level mismatches. Third, residual EEG noise or subject-specific variability may further affect prediction stability. In addition, the number of subjects with severe depression is relatively limited, which may reduce the model's ability to learn robust representations for this subgroup. Future improvements may include enlarging the sample size, improving artifact suppression, and incorporating subject-robust regression strategies to further enhance prediction performance for difficult cases.

For the majority of subjects, the predicted values align closely with the depression levels of the true values, particularly in the case of intermediate-level depression. Patients at these depression levels may heavily rely on a doctor's clinical judgment for diagnosis in practice. Consequently, this model can effectively aid doctors in initial depression screening. However, it's important to acknowledge that the model's predictive ability diminishes for patients with severe depression, potentially necessitating further evaluation by a medical professional. Here is the polished version of the paragraph:

At a broader scope, using moderate depression as the delineating threshold, the model demonstrates high proficiency in predicting mild depression levels below the moderate range. Depression falling below the moderate level in the PHQ-9 self-assessment questionnaire can be categorized as subthreshold depression, which typically manifests in the early phases of depressive disorders, particularly when symptoms are not yet manifestly evident (Rodríguez et al., 2012). Consequently, this model holds the potential to furnish effective subthreshold diagnoses, enabling the timely implementation of treatment or intervention measures to forestall further progression or exacerbation of the condition.

**Figure 6:**
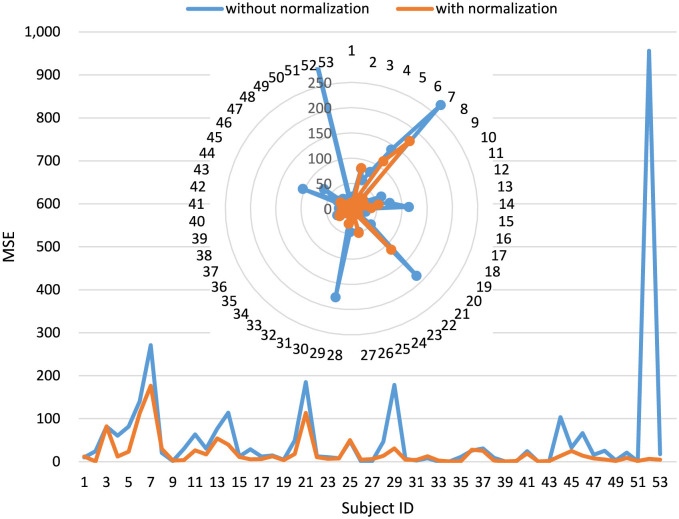
Comparison between the performance of MCEEGNet for regression testing with and without label normalization. In both the main panel and the magnified inset, the blue line denotes the results obtained without label normalization, while the orange line denotes the results obtained with label normalization.

Figure 6 compares the regression performance of MCEEGNet with and without label normalization. In both the main panel and the magnified inset, the blue curve represents the results obtained without label normalization, whereas the orange curve represents the results after applying label normalization. On the x-axis, different subjects or samples are represented, while the y-axis displays the MSE values, which serve as a measure of prediction accuracy. The figure displays two distinct lines: one in blue and the other in orange. The blue line signifies the results obtained without label normalization, while the orange line represents the results obtained after applying label normalization.

Key findings from this comparative analysis are as follows: (1) The orange line (with label normalization) consistently exhibits lower MSE values compared to the blue line (without label normalization), indicating a positive impact of label normalization on the model's performance in regression testing. (2) Subjects or samples with smaller label values, such as subjects 29 and 52, tend to benefit more from label normalization, as evidenced by the larger discrepancy between the two lines for these samples. (3) In general, label normalization enhances the model's ability to make predictions that closely align with the true target values, particularly for samples with diverse label value ranges. This analysis underscores the effectiveness of label normalization as a technique for improving the accuracy of regression predictions using MCEEGNet. It ensures that the model can adeptly handle target variables with varying value ranges, ultimately resulting in more precise and dependable predictions.

Depression severity is typically assessed using the PHQ-9 scale test. Given that the evaluation metrics of the PHQ-9 span from 0 to 27, there can be significant variations and differences in individual scores. Consequently, when conducting regression predictions, normalizing the data labels through label scaling or target variable scaling proves beneficial in enhancing model performance and training effectiveness.

**Figure 7:**
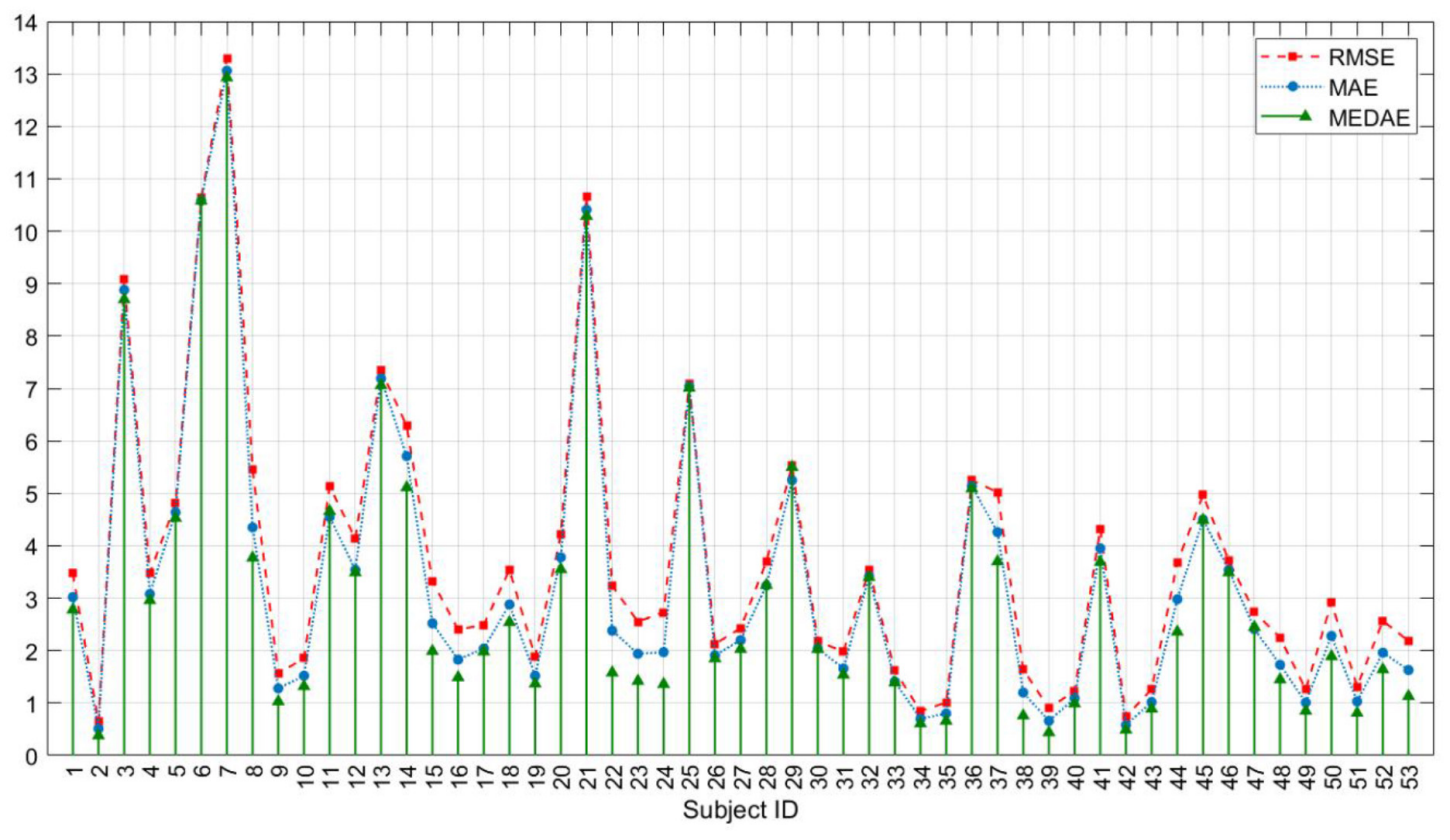
Predictive scores for all subjects in regression test by MCEEGNet.

Furthermore, the study includes additional metrics to assess the prediction results for each subject, specifically RMSE (Root Mean Square Error), MAE (Mean Absolute Error), and MEDAE (Median Absolute Error), as illustrated in Figure 7. When analyzing the overall performance across all subjects, it is evident that, except subjects 6, 7, and 21, all other subjects exhibit values below 10 for all of these metrics. Smaller values for RMSE, MAE, and MEDAE are indicative of more precise predictions. Notably, subjects 2, 34, 35, 39, and 42, among others, demonstrate particularly high prediction accuracy, with RMSE values consistently below 1.

Regarding the distribution of errors, certain subjects exhibit relatively larger RMSE and MAE values, while their MEDAE values remain small. This observation implies the influence of outliers on the RMSE and MAE results. For the majority of subjects, the MEDAE is notably smaller than both MAE and RMSE, underscoring the model's relative accuracy in predicting the majority of data points.

To gain a comprehensive understanding of the capabilities, limitations, and potential contributions of the proposed model, we conducted a comparative analysis of predictive results between our model and established machine learning techniques, all utilizing the same dataset and protocol. The results are presented in Table 3. The methods under scrutiny encompass Linear Regression, Decision Tree, ExtraTree, AdaBoost, k-Nearest Neighbors (KNN), Bagging, Gradient Boosting, Random Forest, and Support Vector Machine (SVM) (Pedregosa et al., 2011). All these approaches were executed with consistent protocols and label normalization. It is worth noting that some baseline methods, especially Linear Regression, show a standard deviation larger than the mean MSE, these statistics were computed from subject-wise regression errors. Because MSE squares residuals, a small number of subjects with large prediction errors can substantially inflate both the mean and the standard deviation, resulting in a highly skewed distribution. By contrast, MAE and MEDAE are less sensitive to outliers, which explains their relatively smaller dispersion.

**Table 3:** Comparison of regression performance of existing methods and the proposed method.

Method	MSE	RMSE	MAE	MEDAE
Linear regression	156.68 / 259.58	10.49 / 6.89	9.34 / 5.24	8.44 / 4.73
ExtraTree	143.23 / 86.05	11.21 / 4.22	9.45 / 4.15	9.33 / 6.34
Decision tree	141.09 / 97.26	11.20 / 3.99	9.32 / 4.24	8.88 / 6.02
AdaBoost	111.53 / 98.92	9.28 / 5.08	8.33 / 5.24	8.49 / 6.35
KNN	91.38 / 81.36	8.37 / 4.66	8.22 / 4.69	8.30 / 4.76
Bagging	87.40 / 79.19	8.31 / 4.33	7.92 / 4.42	7.87 / 4.51
Gradient boosting	83.56 / 72.17	8.24 / 3.99	7.81 / 4.11	7.93 / 4.42
Random forest	79.49 / 68.09	7.98 / 4.02	7.72 / 4.12	7.78 / 4.29
SVM	75.84 / 61.85	7.93 / 3.64	7.93 / 3.64	7.93 / 3.64
MCEEGNet (without normalization)	56.01 / 135.77	5.50 / 5.13	5.22 / 5.10	5.10 / 5.09
MCEEGNet (ours)	20.45 / 2.7	3.66 / 2.68	3.28 / 2.70	3.07 / 2.74

For each metric, values are reported as mean ± standard deviation across subjects.

Additionally, we included the proposed model without label normalization to facilitate a comprehensive comparison. Table 3 unmistakably illustrates that the proposed model outperforms all the comparative methods, yielding notably superior results across all four metrics. Even the MCEEGNet model without label normalization exhibits substantial performance gains compared to the alternative methods, underscoring the robustness of the MCEEGNet model. These findings contribute significantly to the advancement of a more potent, resilient, and practically valuable model for the diagnosis of depression.

## 4 Conclusion

This study proposes a novel Multi-Cue EEG neural network for depression diagnosis, which can simultaneously realize classification identification and regression prediction of depression. The method processes prefiltered EEG signals corresponding to various stimuli through distinct branches of MCEEGNet to extract features that capture shared depression patterns across different cues. It demonstrates strong performance in automating depression diagnosis, particularly in diagnosing subthreshold depression. Multi-cue recognition in EEG-based depression diagnosis can be extended to encompass multi-source stimuli like light, sound, and video, thereby offering a more comprehensive and precise elucidation of depression biomarkers. The proposed framework holds significant potential for identifying individuals with depression, and it is strongly recommended that the medical device industry leverage this framework in the development of diagnostic systems for depression.

## Data Availability

The original contributions presented in the study are included in the article/supplementary material, further inquiries can be directed to the corresponding author.

## Footnotes

1
http://modma.lzu.edu.cn/data/application/#data_1

2
https://sccn.ucsd.edu/eeglab/index.php
